# Glances at the Hospitals

**Published:** 1900-08-25

**Authors:** 


### GLANCES AT THE HOSPITALS.

### THE TORBAY HOSPITAL.

This institution had under treatment during 1899 a total of 359 in-patients. Of these 256 were discharged recovered, 42 relieved, 8 incurable, 10 at their own request, 17 died, and 26 were under treatment on the date of the report. There is a provident dispensary attached to the hospital. During the year 214 new members joined and 224 were removed from the books. The board regret that so many wait to join until illness overtakes them, instead of looking forward and providing for it; and a new rule has been added which, it is hoped, will check this lack of forethought on the patients' part. In the ophthalmic department 518 patients were treated in the out-patients' department, and altogether 89 operations were performed in the eye department, 31 of them being for cataract. A table of the other surgical operations is not given. The total income was £2,074, and the total expenditure was £2,127.

### THE ROYAL ALBERT HOSPITAL AT DEVONPORT.

The total cost of maintenance in this hospital was £4,375, as compared with £4,248 in the previous year. There was an adverse balance of £487, but this has been reduced to £239; not, however, owing to any increase of the annual subscriptions. A bazaar produced £717, and a pastoral play £75. Valuable as these expedients are at exceptional times, it is clear that they cannot be relied on as a regular source of income, and if the hospital is to be kept free from debt it must either be by an increase in the number of subscribers or a decrease in the number of beds occupied. We are always glad to be able to chronicle the efforts of the workpeople themselves to add to the income of any charity of which they are the chief participants, and here the factory and dockyard hands contributed £183. At the beginning of the year the hospital contained 69 patients; 821 were admitted during the year, and 63 remained under treatment at the end of it. The statistics show that 667 were discharged recovered, 81 relieved, 41 left for other reasons, and 38 died. The average daily number resident was 69, the mean residence of each patient was 28½ days, and the mortality was 4·27 per cent. of the total number under treatment. The medical tables are good so far as they go, but that which states the nature of the operations performed does not give the results of these operations, and it is in some cases impossible to extract the information we want from the general tables. For example, we find that 34 operations were performed for cataract, and on referring to the ophthalmic table we find that 44 cases of cataract had been treated, and there our information ends; but if we look at the figures on page 28 we then find that 25 cataract cases recovered, one was relieved, and one was not relieved. All this is no doubt plain enough to those who made out the statistics, but it is hardly clear to the ordinary reader. Again, the table of operations tells us that the operation for the radical cure of hernia was performed 16 times, and that for strangulated hernia six times. The medical report says there were 29 cases of hernia, of whom 23 recovered, two were relieved, two not relieved, and one died.

